# Leukotrienes in Atherosclerosis: New Target Insights and Future Therapy Perspectives

**DOI:** 10.1155/2009/737282

**Published:** 2010-01-26

**Authors:** Graziano Riccioni, Alessandra Zanasi, Nicola Vitulano, Barbara Mancini, Nicolantonio D'Orazio

**Affiliations:** ^1^Cardiology Unit, “San Camillo de Lellis” Hospital, Manfredonia, Foggia, Italy; ^2^Cardiology Unit, University of Foggia, Foggia, Italy; ^3^Cardiology Unit, Catholic University, Roma, Italy; ^4^Department of Biomedical Sciences, University of Chieti, Italy

## Abstract

Atherosclerosis represents an important chronic inflammatory process associated with several pathophysiological reactions in the vascular wall. The arachidonic acid, released by phospholipase A2, is an important substrate for the production of a group of lipid mediators known as leukotrienes, which induce proinflammatory signaling through the activation of specific BLT and CysLT receptors. The interaction of these substances in the vascular wall determines important morphological alterations like the early lipid retention and the accumulation of foam cells, the development of intimal hyperplasia, and advanced atherosclerotic lesions, and it plays an important role in the rupture of atherosclerotic plaque. Many studies regarding myocardial ischemia and reperfusion show that leukotriene signaling may be involved in the development of ischemic injury. For these, reasons both leukotriene synthesis inhibitors and leukotriene receptor antagonists have been suggested for inducing beneficial effects at different stages of the atherosclerosis process and may represent a new therapeutic target in the treatment of atherosclerotic vessel diseases, in particular in acute coronary syndrome.

## 1. Introduction

Cysteinyl leukotrienes (Cys-LTs) are potent inflammatory lipid mediators derived from the 5-lypoxygenase (5-LO) pathway of arachidonic acid (AA) metabolism [1], initially identified to have important effects on pathogenetic aspects of allergic rhinitis and bronchial asthma and approved in the late 1990s for the relief of perennial and seasonal allergic rhinitis symptoms, and the treatment of mild persistent bronchial asthma [2–5].

 Recently many studies revealed the presence of Cys-LTs in atherosclerotic lesions playing a key role as signaling molecules in atherosclerosis [6, 7], abdominal aneurysms [8, 9], intimal hyperplasia [10], and with possible effects on tumorigenesis [11, 12]. For these reasons, the leukotriene pathway may represent an alternative therapeutic target in the treatment of atherosclerotic vessel diseases [6, 13, 14].

## 2. Biochemistry of Endothelium

Vascular endothelium is an active endocrine, paracrine, and autocrine organ, indispensable for the maintenance of vascular homeostasis [15]. When it is altered by various stimuli, it may cause localized alterations or “*endothelial dysfunction*” having antihemostatic properties, regulating vascular tone, determining a heightened leukocyte adhesion, and increase production of cytokines and growth factors [16]. The term “*endothelial activation*” designates a subset of endothelial dysfunction whereby some changes produced by various stimuli elicit new functional and molecular properties. The endothelium activation contributes to the regulation of vascular tone, haemostasis, and blood leukocyte recruitment and determines the releasing of vasodilators like nitric oxide [17] and prostacyclin [18], and vasoconstrictors such as endothelin [19] and platelets activating factor [20].

## 3. Biochemistry of Leukotrienes: Mediators and Receptors

The term “*eicosanoids*” includes prostaglandins (PGs), tromboxanes (TX), leukotrienes (LTs), and hydroxyl-eicosatetraenoic acid made by polyunsaturated 20-carbon fatty acids (PUFA), including the most abundant and biological precursor AA [21]. The AA, a normal component of cell membrane phospholipids, serve as substrate for prostaglandin endoperoxide (PGH) syntases-1 and -2, also known as cycloxygenase (COX)-1 and -2, lipoxygenase (5-, 12-, or 15) (LO), or cytochrome p450 enzymes [18]. 

 LTs exert their biological effects by activating specific receptors belonging to the superfamily of G protein-coupled receptors (GPCRs) [22–24]. Two receptors for LTB_4_ have been molecularly identified: BLT1 and BLT2. BLT1 is a high-affinity receptor specific for LTB_4_, which is expressed primarily in leukocytes and mediates chemotaxis [25]; BLT2 is a pharmacologically distinct receptor, which is ubiquitously expressed and displays low affinity for LTB4 and also binds other eicosanoids [26, 27]. The ubiquitous expression and the broader ligand specificity suggest that BLT2 may mediate distinct biological and pathophysiological roles from BLT1. 

**Figure 1:**
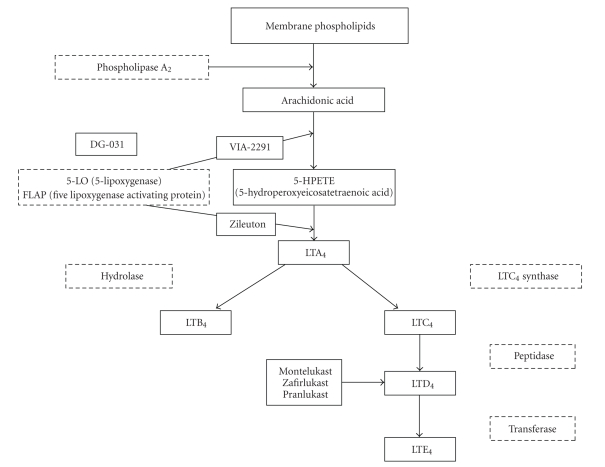
Leukotrienes biosynthesis scheme.

 Receptors responding to Cys-LTs have been cloned in 1999 and termed CysLT1 and CysLT2 [22]. CysLT1 recombinant receptor is activated by all the native ligands, with a rank-order potency of LTD4 > LTC4 > LTE4 [28, 29], whereas for CysLT_2_ receptor the agonist rank order potency is LTD_4_ = LTC_4_ with LTE_4_ less potent [30–32]. Despite the classic view that the activity of cysteinyl-LTs is due to the interaction with these two specific plasma membrane receptors, alternative pathways have been postulated, including localization of CysLT receptors at nuclear level, cross talk with other membrane receptors, the possibility that CysLT receptors might exist as homo/heterodimers, and the existence of additional receptor subtypes [22]. The expression of BLT and CysLT subtypes on vascular smooth muscle and endothelial cells is highly dependent on transcriptional regulation by pro- and antiinflammatory mediators [22, 33] (see Figure 1). 

 Recent experimental evidences in vitro and in vivo suggest that all the responses can be attributed to the existence of additional subtypes and/or to the formation of homo- and/or heterodimers [34, 35]. Few reports suggest that CysLTs receptors could also be localized as levels other than on the plasma membrane, suggesting an important and unanticipated role for these receptors in cell signalling and function [36]. Some observations are stimulating research towards the potential roles of the 5-LO pathway in promoting inflammation in cardiovascular disease, including atherosclerosis and aortic aneurysms [37] (Table 1). 

 A major role for the LT pathway in vascular disease was suggested by studies of a congenic mouse strain demonstrating that atherosclerosis resistance is linked to a locus on chromosome 6, in which the gene for 5-LO was mapped subsequently [38] (Table 1). 

 Additional evidence comes from the identification of enzymes and receptors in the 5-LO pathway expressed in human atherosclerotic plaques [39] and in subjects with atherosclerosis as measured by an increased intima-media thickness [40]. Moreover, variants in the FLAP encoding gene have been associated with an increased risk in humans [41]. 

 Therefore, multiple lines of evidence including in vitro studies, animal models (mice and mouse strains, rat), and some human studies implicated LTs in the pathogenesis of atherosclerosis, and in particular suggested an important role for LTB_4_ and BLT receptor in atherogenic processes [8, 42, 43].

## 4. LTs Modifiers: Receptor Antagonists

In order to regulate the effects of Cys-LTs pharmacologically, two general approaches have been used with success: LTs synthesis inhibition and LTs receptor antagonism. The LT synthesis inhibitor blocks key steps in the biosynthetic pathway (either 5-LO or 5-LO activating protein—FLAP) to inhibit production of both Cys-LTs and LTB_4_, whereas the LTRAs selectively block the CysLT_1_ receptor on target cells. Pharmaceutical agents such as montelukast, zafirlukast, and pranlukast are well tolerated and have been approved in the USA, Europe, and other areas for the treatment of allergic rhinitis and bronchial asthma [4, 5].

## 5. LTs Modifiers: 5-LO Synthesis Inhibition

The first committed step in the synthesis of LTs is the oxidation of AA by 5-LO, and the integral membrane protein FLAP is an essential partner of 5-LO for this process [44]. FLAP was molecularly identified through a photoaffinity probe and an affinity gel based on MK-886, a selective leukotriene inhibitor that has no activity against broken-cell preparations of 5-LO [45]. Several FLAP inhibitors showed efficacy in early clinical trials in asthma but were not developed commercially for unpublished reasons [46]. 

 Zileuton (ZIL) is the only marketed drug with a specific effect on Cys-LTs synthesis through the inhibition of the 5-LO enzyme, administered orally four times daily (QID). It is metabolized by the cytochrome P450 isoenzymes and may therefore interact with other drugs metabolized by these enzymes, such as theophylline and warfarin [47, 48]. The use of ZIL is hampered because it needs a QID dispensing and the monitoring of liver enzymes [49]. It is approved for the treatment of asthma in 12-years-old patients and older ones [48].

## 6. LTs Modifiers in the Treatment of Cardiovascular Disease

Atherosclerosis is an inflammatory process associated with several pathophysiological reactions within the vascular wall [50–52]. LTs have not been perceived as promoters of CVD until recently, despite actions of LTs in the cardiovascular system (CVS). 

 The connection originated from mouse genetic studies, where a locus on chromosome 6 was identified to confer almost total resistance to atherogenesis [53]; further analysis identified 5-LO as a major gene contributing to atherosclerosis susceptibility in mice [54]. 5-LO was found to be present in human atherosclerotic aorta, coronary and carotid arteries [55] and variant 5-LO genotypes identified a subpopulation with increased atherosclerosis [56]. Also studies on FLAP and LTA_4_ hydrolase support involvement of LTs in human atherosclerosis [57–59]. The importance of the link between 5-LO and atherosclerosis has been exhaustively reviewed [59–61]. However, a recent quantitative analysis of atherosclerotic lesions in 5-LO^−/−^ mice seems to argue with a role of the 5-LO pathway in intermediate to advanced atherosclerotic lesion development [62]. 

 LT products of 5-LO are primarily produced by monocyte macrophages in the arterial intima, foster the chemoattraction of monocytes, T-cells, or other types of circulating cells within the vessel wall, increase vascular permeability, or both through activation of LT receptors. Following are the circumstantial and experimental evidence supporting a link between LTs, their receptors, and atherosclerosis.

### 6.1. DG-031 (BAY × 1005)

DG-031, formerly known as BAY × 1005, acts by binding the FLAP protein and preventing the translocation of 5-LO from the citosol to the cell membrane. This drug both competes for binding sites on the cell membrane with 5-LO and is a functional competitive inhibitor of FLAP. deCODE genetics has linked variants in the gene encoding FLAP and the gene encoding leukotriene A_4_ hydrolase (LTA_4_H) to the risk of heart attack. These variants appear to confer increased risk of heart attack by increasing the production of LTB_4_, a potent driver of inflammation produced in atherosclerotic plaques. 

 Hakonarson et al. [63] conducted a randomized, placebo-controlled, crossover trial about DG-031 on 268 patients and found a significant dose-dependent suppression of inflammatory biomarkers (LTB_4_ and myeloperoxidase) associated with the risk of MI. In this study, patients were first randomized to receive 250, 500, and 750 mg/daily of DG-031 or placebo for 4 weeks of treatment after a 2-week wash out period.

### 6.2. VIA-2291

Recently have been announced the results of Phase 2 trials in acute coronary syndrome (ACS) investigating VIA-2291, a selective and reversible inhibitor of 5-LO, a key enzyme in LTs biosynthesis. The ACS Phase 2 study of VIA-2291 was designed to establish optimal dosing and safety data in 191 patients with ACS, who recently had a heart attack or unstable angina. Patients were treated once daily for 12 weeks with one of three doses of VIA-2291 or placebo. In order to further evaluate VIA-2291's effect over a longer timeframe, a substudy of patients in the ACS trial continued for additional 12 weeks of treatment at the same dose followed by a 64-slice multidetector computed tomography (MDCT) scan following up on the baseline MDCT scan that all patients had received. The statistical outcomes for the ACS trial were validated by an independent academic statistics group at Montreal Heart Institute. The ACS trial demonstrated a statistically significant, dose-dependent inhibition of ex vivo-stimulated LTB4 production at 12 weeks. LTB4 production was measured at trough, just before the next dose of VIA-2291 was taken, indicating a sustained pharmacological effect of the drug between doses. The secondary endpoint of change from baseline in urine LTE4, also showed significant inhibition at all dose levels. The drug was generally well tolerated in the trials. Common (>10 percent) adverse events with no clear difference between placebo and VIA-2291 treated patients included angina, fatigue, musculoskeletal pain, and headache. Laboratory abnormalities included generally mild, reversible ≥ 3x upper limit of normal liver enzymes in the low-dose VIA-2291 treated group (10 percent) and placebo (2 percent), not seen in the higher-dose drug-treated groups, and asymptomatic ≥ 1.5x lipase elevations that were more common in VIA-2291 treated patients [44].

## 7. Conclusion

LTs are potent inflammatory mediators synthesized within the cardiovascular system through the 5-LO pathway of AA metabolism. CysLTs are vasoconstrictors and induce endothelium dependent vascular response through the Cys-LT1, and Cys-LT2 receptor subtypes. Taken together, experimental and genetic studies suggest a major role of LTs in atherosclerosis and in its ischemic complications such as acute coronary syndromes and stroke [64]. 

 Furthermore, the effects on vascular smooth muscle cells suggest a role in the vascular remodeling observed after coronaric angioplasty, as well as in aortic aneurysm. Further experimental and clinical studies are needed to determine the potential therapeutic strategies targeting the LT pathway in cardiovascular disease [65, 66]. Recent evidences regarding the application of LT modifiers greatly increased a potential use of these drugs in cardiovascular and cerebrovascular diseases. The exact role of LTRAs in disease management is still evolving. Large-scale, controlled trials are needed to determine the effectiveness and the safety deriving from the use of LTRAs in cardiovascular diseases [67].

## 8. Financial and Computing Interests

The authors have no relevant affiliations or financial involvement with any organization or entity with a financial interest in or financial conflict with the subject matter or materials discussed in the manuscript. This includes employment, consultancies, honoraria, stock ownership or options, expert testimony, grants or patents received or pending, or royalties. No writing assistance was utilized in the production of this manuscript.

## Figures and Tables

**Table 1:** Step of biosynthetic pathway, site of action, and blocking agents.

Step of biosynthetic pathway	Site of action	Blocking agent
Flap (five-lipoxygenase activating protein)	Cell membrane	Not developed commercially
5-LO (5-lipoxygenase)	Citosol	Zileuton
	Citosol	Via-2291
Translocation of 5-lipoxygenase	Binding sites	DG-031 (BAY × 1005)
	Cell membrane	
CysLT_1_ receptor	Target cells	Montelukast
		Zafirlukast
		Pranlukast
